# Preparation of Cinnamon Essential Oil/Succinic Acid-Modified Cyclodextrin and Their Application in Grass Carp Preservation

**DOI:** 10.3390/foods14234100

**Published:** 2025-11-29

**Authors:** Xiaoshan Li, Haoxin Li, Yuemei Zhang, Wendi Teng, Ying Wang, Jinxuan Cao, Jinpeng Wang

**Affiliations:** 1 Key Laboratory of Geriatric Nutrition and Health, Beijing Technology and Business University, Ministry of Education, Beijing 100048, China; lixiaoshan4321@163.com (X.L.); 1430528220@163.com (H.L.); zhangyuemei@btbu.edu.cn (Y.Z.); wenditeng@btbu.edu.cn (W.T.); wang-ying@btbu.edu.cn (Y.W.); caojinxuan@btbu.edu.cn (J.C.); 2 School of Food and Health, Beijing Technology and Business University, Beijing 100048, China; 3 College of Food and Biological Engineering, Chengdu University, Chengdu 610106, China

**Keywords:** cyclodextrin carboxylate, grass carp, preservation, cinnamon essential oil

## Abstract

The spoilage of grass carp is driven by multiple factors, predominantly microbial proliferation and lipid oxidation. Although cinnamon essential oil exhibits potent antibacterial and antioxidant properties, its industrial application is constrained by high volatility, poor water solubility, and a strong pungent odour. To address these challenges, β-cyclodextrin-succinate (SACD) was synthesized via esterification. Prior studies confirmed that SACD demonstrates significantly improved solubility and antibacterial efficacy compared to β-CD. Thereafter, SACD was employed to encapsulate cinnamon essential oil. Moreover, the succinic acid-modified cyclodextrin-encapsulated essential oil exhibits a stronger antioxidant capacity compared to the free essential oil. Through a series of characterization techniques and molecular docking analysis, the successful synthesis of SACD and its inclusion complexes was confirmed. To assess their efficacy in preserving grass carp meat, four experimental groups were established: a control group, an essential oil group (EO), an unmodified inclusion complex group (EO/CD), and a modified inclusion complex group (EO/SACD). Under refrigerated conditions at 4 °C, the pH, drip loss rate, TBARS, TVC, TVB-N, K-value, and texture of fish meat samples were determined across different treatment groups. During the later stages of storage, the total bacterial count in the inclusion complex group was lower than that in the EO group and the control group, indicating that the cyclodextrin-encapsulated essential oil effectively inhibited microbial growth (*p* < 0.05). The results demonstrated that the EO/SACD group delayed spoilage and prolonged the storage period of grass carp compared to both the EO and EO/CD groups.

## 1. Introduction

Grass carp is a species of the genus Ctenopharyngodon (family Cyprinidae, order Cypriniformes) and one of the Four Major Carp Species in Chinese freshwater aquaculture [1]. Its high water content, combined with rich unsaturated fatty acids, high endogenous enzyme activity, and neutral pH, accelerates spoilage, making processing, storage, and transport challenging [2]. Although enzymatic processes and lipid oxidation are involved in spoilage, microbial growth is generally the predominant factor responsible for generating harmful compounds and offensive odors [3]. Therefore, effective preservation techniques are essential to prevent quality degradation, extend shelf life, and reduce economic losses while enhancing edible value. Current methods for aquatic products include low-temperature storage, controlled atmosphere preservation, ultra-high pressure processing, and biopreservation [4]. In light of growing public concern over synthetic preservatives, the development of safe, effective, and naturally derived preservatives has become a critical priority for the grass carp industry [5]. Cinnamon essential oil is a natural additive with broad-spectrum antimicrobial activity, widely utilized in pharmaceuticals, food seasonings, cosmetics, beverages, household fragrances, and the chemical industry [6]. It is recognized as ‘generally recognized as safe’ and is considered to have enormous application potential in the food industry [7]. Numerous studies have demonstrated its significant antimicrobial efficacy against pathogenic bacteria [8]. However, its application is limited by strong volatility, photothermal instability, and susceptibility to oxidation [9]. The development of stabilization techniques is crucial for enhancing the commercial viability of essential oils. To address these limitations, microencapsulation technology has been widely adopted to improve EO stability and expand its applications.

Cyclodextrins are widely employed as wall materials for microencapsulation, and their production technology is well-established. As cyclic oligosaccharides, their structure comprises a hydrophilic exterior and a hydrophobic internal cavity. This unique architecture represents their most defining characteristic [10]. It enables cyclodextrins to function as host molecules, encapsulating various guest substances within their cavity to form stable inclusion complexes. This process confers multiple benefits: enhanced stability against heat, light, and oxidation; improved aqueous solubility of poorly water-soluble guests; and controlled release kinetics [11]. In a previous study, encapsulation of Art within β-CD significantly improved its aqueous dispersibility, leading to a marked increase in its antibacterial efficacy against methicillin-resistant *Staphylococcus aureus* compared to the unencapsulated form [12]. However, the low solubility of β-CD limits its application in hydrophilic systems. To address this, chemical modifications of natural CDs have been developed to enhance water solubility and biological functionality. Several cyclodextrin derivatives—Several modified β-cyclodextrin derivatives—including hydroxypropyl-β-cyclodextrin (HP-β-CD), sulfobutyl ether-β-cyclodextrin (SBE-β-CD), and methyl-β-cyclodextrin (M-β-CD)—have been developed and are currently used in pharmaceutical formulations [13,14]. Furthermore, structural modifications can introduce specialized properties. For example, grafting octenyl succinic anhydride onto α-cyclodextrin yields amphiphilic derivatives with enhanced emulsification capacity [15]; additionally, succinic anhydride-modified γ-cyclodextrin has been employed in the development of fish meat preservation agents [16].

This study developed an advanced encapsulation system using synthesized SACD to alleviate the volatility and poor solubility of cinnamon EO, thereby enhancing its efficacy in extending the shelf life of grass carp. The SACD carrier itself exhibits superior solubility and antimicrobial properties, and the resulting EO/SACD complex demonstrated effective preservation capabilities.

## 2. Materials and Methods

### 2.1. Screening Essential Oils

The minimum inhibitory concentration (MIC) of essential oils against four bacterial species—*Escherichia coli*, *Staphylococcus aureus*, *Listeria monocytogenes and Pseudomonas* (Ningbo testobio Co., Ltd., Ningbo, China)—was determined using a microdilution gradient approach. Briefly, 100 μL of each EO (Oregano EO, cinnamon bark EO, thyme EO, and lemongrass EO) (Shanghai Yuanye Biotechnology Co., Ltd., Shanghai, China) was diluted with culture medium to form a series of double-concentration gradients. The prepared bacterial suspension (100 μL) was added to each well and thoroughly mixed. Wells containing an equal volume of bacterial suspension were designated the positive controls, and those without the suspension were designated the negative controls. After incubation at 37 °C for 24 h, the MIC was defined as the lowest concentration yielding a clear well [17].

### 2.2. Modification of β-Cyclodextrin

The sample was prepared by mixing 10 g of β-CD, 8 g of succinic acid, 10 g of sodium hypophosphite (Shanghai Yuanye Biotechnology Co., Ltd., Shanghai, China), and 100 mL of deionised water with continuous stirring until complete dissolution. The mixture was first oven-dried at 105 °C. After the initial drying, the sample was further heated at 135 °C for a duration of 20 min. The crude product was then dissolved in water, washed with anhydrous ethanol, and separated. The final purified product was obtained by drying overnight at 50 °C [18].

### 2.3. Preparation of Inclusion Compounds

The EO/SACD complex was prepared according to established methods [19]. Briefly, 1.5 g of SACD was dissolved in 25 mL of distilled water under magnetic stirring at 650 rpm and room temperature. A solution of cinnamon EO in ethanol (150 μL EO: 600 μL ethanol, 1:4 *v*/*v*) was added dropwise to the aqueous CD solution. The mixture was sealed, protected from light, and stirred continuously at 37 °C for 12 h. The resulting product was freeze-dried for 72 h (Nanjing Jinshi Eco-mini Basic Laboratory Freeze Dryer, Nanjing, China), washed three times with anhydrous ethanol to remove unencapsulated EO, and air-dried at room temperature. The final EO/SACD complex powder was packaged in sealed plastic bags and stored in a desiccator for subsequent use [20].

### 2.4. Determination of Encapsulation Rate

After washing with anhydrous ethanol to remove surface oil, the inclusion compound was dissolved in fresh anhydrous ethanol, sonicated for 5 min, and centrifuged at 8000 rpm for 8 min. The absorbance of the supernatant was measured, and this value was applied to the standard curve to calculate the EO concentration [21]. The inclusion efficiency (IE) was calculated using the formula:

(1) $$\mathrm{IE}\;(\%)\;=\;\frac{M_{1}}{M_{2}}\;\times \;100\%$$

where M_2_ is the total amount of EO added (g); M_1_ is the mass of EO in the complex (g).

### 2.5. Structural Characterisation of Modified Cyclodextrins and Inclusion Compounds

#### 2.5.1. Fourier Transform Infrared Spectroscopy (FTIR)

The FTIR spectra of SA, β-CD, SACD, EO, and the EO/SACD complex were obtained using a Nicolet Summit FTIR spectrometer (Thermo Fisher Scientific, Shanghai, China). Using a pellet of approximately 2 mg of sample, the spectrometer was used to record spectra across the 400–4000 cm^−1^ spectral range, accumulating 32 scans per measurement.

#### 2.5.2. Nuclear Magnetic Resonance

Sample preparation for ^1^H NMR involved accurately weighing approximately 0.02 g, dissolving it in D_2_O, and sonicating for 10 min. The resulting solution was then transferred into an NMR tube for ^1^H NMR analysis, which was performed on a spectrometer (Bruker AVANCE III, 400 MHz, Bruker Corporation, Ettlingen, Germany). The degree of substitution (DS) of SACD was calculated based on the ratio of the proton peak integration of the methylene (-CH_2_) group in the SA moiety to that of β-CD.

#### 2.5.3. X-Ray Diffraction Testing

Crystallographic analysis was conducted by X-ray diffraction (Bruker D8 ADVANCE X-ray Diffractometer, Bruker Corporation, Germany). The dried test sample was fixed on a glass slide for testing. The measurement conditions were as follows: employing Cu-Kα radiation (λ = 1.543 Å) with the source operated at 40 kV and 40 mA, and scanning over a 20 range of 4° to 40°.

#### 2.5.4. Thermogravimetric Analysis

The thermal properties of the sample were characterized using a TGA2 thermal analysis system (NETZSCH STA 449 F3/F5, Nerz Instruments Manufacturing GmbH, Troisdorf, Germany). TGA measurements were performed on precisely weighed samples (3–5 mg) using a standard ceramic crucible. Thermogravimetric analysis was employed under a flowing N_2_ environment (50 mL/min), applying a linear heating ramp from 30 to 600 °C at 10 °C/min.

#### 2.5.5. Electron Microscope

After being mounted on a sample stage with conductive adhesive, the specimen was subsequently gold-sputtered to enhance surface conductivity. Its microstructure was then examined by SEM (Hitachi SU8020, Hitachi Limited, Tokyo, Japan) operating at an accelerating voltage of 5 kV.

### 2.6. Antioxidant Activity

The antioxidant activity was determined using the DPPH assay, following the employed protocol [22]. Test solutions (1 mL; EO/SACD, EO, or ascorbic acid at 0.1–2 mg/mL in ethanol) were reacted with 1 mL of 0.1 mM DPPH ethanol solution. After vortexing, the mixtures underwent a 1 h incubation in darkness at 26 °C. The absorbance at 517 nm was measured, using ascorbic acid as a standard. The percentage inhibition, based on triplicate measurements, was calculated using the formula:

(2) $$\mathrm{DPPH}\;\mathrm{radical}\;\mathrm{scavenging}\;\mathrm{activity}\;\left(\%\right)\;=\;\left[\;\frac{{(A}_{0}\;-\;A_{1})}{A_{0}}\;\right]\times \;100\;$$

where A_0_ represents the absorbance of the blank group, and A_1_ corresponds to the absorbance of the experimental sample.

### 2.7. In Vitro Release Study

The sustained release properties of the inclusion complex were evaluated using a dialysis method. Specifically, 200 mg of inclusion complex was dissolved in buffered saline (PBS, pH 7.4) and evenly distributed into three dialysis bags. The bags were then transferred into a beaker containing 70 mL of deionized water as the dialysate and incubated under constant rotation at 400 rpm and 37 °C. At set intervals, 2 mL of dialysate was sampled and the absorbance was measured spectrophotometrically at 236 nm to determine the sample concentration. The cumulative release percentage was calculated using the following equation [23].

(3) $$\mathrm{Cumulative}\;\mathrm{release}\;\mathrm{rate}\;(\%)=\frac{C_{t}}{N_{0}}\;\times \;100$$

Here, Ct is the EO concentration measured at various time points, and N_0_ stands for the initial encapsulation efficiency of EO in the inclusion complex.

### 2.8. Molecular Docking

To investigate the configuration of the inclusion complex, molecular docking analyses were conducted with the AutoDock Tools software (version 1.5.7). The SACD and β-CD molecules and cinnamaldehyde were drawn using ChemDraw Ultra 8.0 software and optimised using Chem3D’s MM2. Molecular docking experiments were conducted using Autodock Vina software (version 1.5.7), employing cyclodextrin as an inflexible rigid receptor and cinnamaldehyde as a flexible, torsionally free ligand, with the optimal binding energy predicted. The grid box was set to 40 × 50 × 44 Å with a spacing of 0.375 Å, sufficiently large to accommodate the cyclodextrin molecule and allow ligand movement. Following ten docking runs, the pose with the optimal binding affinity, as indicated by the lowest computed free energy, was selected as the candidate for all subsequent analyses [24].

### 2.9. Treatments and Storage of Fish

#### 2.9.1. Preservation of Fresh Meat

After scaling and evisceration, the fish (Beijing Wummei Supermarket, Beijing, China) carcasses were cut into uniformly sized portions, thoroughly rinsed, and then portioned into 10-g samples. All fish samples were treated with either pure cinnamon essential oil (EO) or its inclusion complexes (EO/CD or EO/SACD) at an EO-equivalent dosage of 100 mg/kg. A group treated with distilled water served as the control. All samples were then stored at 4 °C for analysis. Three independent replicates (n = 3) were set up for each treatment at each sampling time point. The changes in drip loss rate, pH value, total bacterial count, TVB-N, and TBARS value of fish meat were measured at 0, 2, 4, 6, 8, and 10 days.

#### 2.9.2. Drip Loss Rate Measurement

Drip loss was assessed by storing the processed fish meat on a tray covered with plastic wrap at 4 °C. The release of exudate during storage was accounted for by weighing the samples before processing (M_1_) and after storage (M_2_). The calculation of the drip loss rate, based on the average of multiple measurements [25], was performed as follows:

(4) $$\mathrm{Drip}\;\mathrm{loss}\;\mathrm{rate}\;(\%)\;=\;(\frac{{M_{1}-M}_{2}}{M_{1}})\times \;100\;$$

#### 2.9.3. Determination of pH

During storage, the meat pH was measured in triplicate using a modified method [26]. Briefly, meat samples were homogenized with distilled water (1:10, *w*/*v*), and the mixture was filtered and centrifuged (3500 rpm, 10 min). The pH of the resultant supernatant was measured, and the average value was reported.

#### 2.9.4. Total Viable Count (TVC)

The total viable bacterial count in the meat samples was determined using a modified method [27]. Briefly, a 5 g sample was aseptically transferred to a sterile homogenization bag containing 45 mL of physiological saline and homogenized for 1–2 min to obtain a 1:10 homogenate. The homogenate was serially diluted in physiological saline, and aliquots were plated on PCA medium. After incubation at 30 °C for 24 h, colony counts were performed.

#### 2.9.5. Determination of TBARS

The TBARS value was determined according to the method of [28]. Briefly, a 4.0 g sample was mixed with 35 mL of a solution containing 0.1% EDTA-2Na and 7.5% trichloroacetic acid, and homogenized at 10,000 rpm for 30 s. After shaking for 30 min, the mixture was filtered, and 4 mL of the filtrate was reacted with 4 mL of 0.02 mol/L TBA solution at 95 °C for 40 min. After cooling, the absorbance was measured at 532 nm against a TBA blank. The TBARS value was quantified as mg MDA equivalents per kg of meat using a standard curve constructed with malondialdehyde (MDA).

#### 2.9.6. Determination of TVB-N

The TVB-N content was determined by homogenizing a 5 g (M) fish fillet sample with 75 mL of water. The homogenate was allowed to stand for 30 min, after which 1 g of magnesium oxide was added. The mixture was then distilled using an automatic Kjeldahl analyzer (K9840, Haineng Company, Jinan, China). The liberated ammonia was trapped in boric acid and titrated with a standard hydrochloric acid solution (C), consuming a volume of V_1_ mL. A reagent blank was run in parallel, requiring a volume of V_2_ mL. The titration endpoint was identified by a distinct color change [29]. Calculation of TVB-N content was performed as follows:

(5) $$X\;=\;\frac{(V_{1}-V_{2})\;\times \;C\;\times \;14\;}{M}\;\times \;100\%$$

#### 2.9.7. Determination of K Value

The samples were subjected to extraction of nucleoside degradation products, namely ATP, ADP, AMP, IMP, HxR, and Hx [30]. The concentrations of these compounds in the resulting filtrate were quantified using high-performance liquid chromatography (Agilent Technologies, Beijing, China), after which the K-value (%) was determined based on the following equation.

(6) $$K\;(\%)=[\frac{\left[\mathrm{HxR}]+[\mathrm{Hx}\right]}{\left[\mathrm{ATP}]+[\mathrm{ADP}]+[\mathrm{AMP}]+[\mathrm{IMP}]+[\mathrm{HxR}]+[\mathrm{Hx}\right]}\;]\;\times \;100\%$$

#### 2.9.8. Texture Analysis

Equal volumes of fish meat samples were analyzed by TPA (TA. PORTABLE Portable Texture Analyzer, Suzhou Baoman Precision Instruments Co., Ltd., Suzhou, China) to determine hardness, elasticity, chewiness, and toughness. The tests were performed using a cylindrical probe under full compression mode [31]. Each sample underwent three independent parallel measurements, with the average value computed from the results.

### 2.10. Statistical Analysis

Results are expressed as the mean ± standard deviation derived from three biologically independent experiments (n = 3). For each biological replicate, measurements were conducted in triplicate. We assessed statistical significance by one-way ANOVA with Tukey’s post-hoc test for multiple comparisons, considering *p* < 0.05 as significant. All data analysis was carried out with SPSS (v.19), while graphing was performed using Origin 2021 and GraphPad Prism (version 10).

## 3. Results and Discussion

### 3.1. Screening of Essential Oils and Characterization

#### 3.1.1. Screening Essential Oils

The results of the minimum inhibitory concentration (MIC) tests indicate that cinnamon EO exhibits the strongest antibacterial activity against the four bacterial strains, while thyme EO shows the weakest activity. Oregano EO and lemongrass EO demonstrate moderate antibacterial activity. The MIC values for thyme EO against all four bacterial strains exceed 2.5 mg/mL, as shown in Table 1. Cinnamon EO had a MIC of 1.25 mg/mL against *Escherichia coli* and *Staphylococcus aureus*, and 2.5 mg/mL against *Pseudomonas fluorescens* and *Listeria monocytogenes*. Based on its superior efficacy, cinnamon EO was selected for subsequent studies. Additionally, cinnamon EO has been reported to be more effective than lemongrass EO in inhibiting *Listeria monocytogenes* [32].

#### 3.1.2. Fourier Transform Infrared Spectroscopy (FTIR)

**Figure 1:**
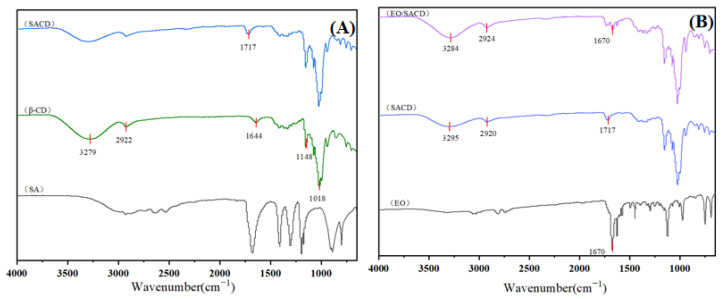
The FTIR (**A**) and (**B**) spectra of SA, β-CD, SACD, EO, EO/SACD.

As shown in Figure 1A, the FTIR spectrum of native β-CD displays characteristic absorption bands at: 3279 cm^−1^ (O-H stretching vibrations), 2922 cm^−1^ (C-H stretching vibrations), 1148 cm^−1^ and 1018 cm^−1^ (C-O stretching vibrations of glycosidic bonds and bound water) [33]. These characteristic peaks remain detectable in SACD, demonstrating that the molecular framework of β-CD is preserved following the dry heat modification process. Notably, SACD exhibits a new absorption band at 1717 cm^−1^, which is assigned to the C=O stretching vibration from ester carbonyl groups [34]. This confirms the successful esterification of β-CD with succinic acid, demonstrating the covalent incorporation of succinic acid into the β-CD framework.

In Figure 1B, the EO spectrum shows a distinct absorption band at 1670 cm^−1^, corresponding to its skeletal functional groups [35]. This peak persists in the EO/SACD complex but with significantly reduced intensity, indicating successful encapsulation of EO within the SACD matrix. The attenuation of this band further supports the formation of an inclusion complex, wherein EO molecules are shielded by the SACD cavity.

#### 3.1.3. Nuclear Magnetic Resonance

**Figure 2:**
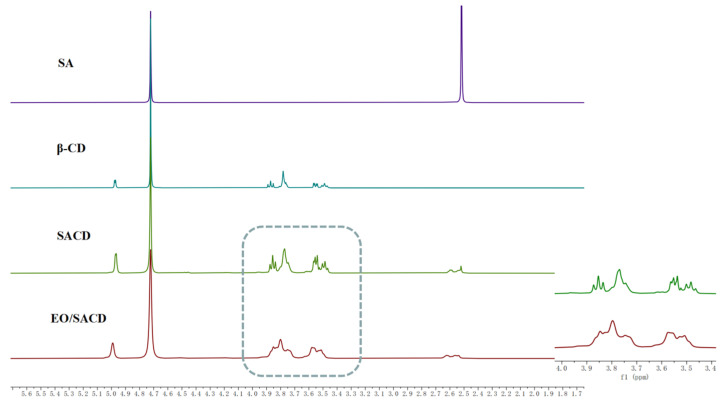
The ^1^HNRM spectra of SA, β-CD, SACD and EO/SACD.

As shown in Figure 2, the proton peak of -CH_2_ in the SA molecule appears at 2.59 ppm, while the proton peaks of β-CD appear at 5.04 ppm (H-1), 3.92~3.96 ppm (H-3), 3.82~3.88 ppm (H-6 and H-5), 3.61~3.63 ppm (H-4), and 3.54~3.58 ppm (H-2) [36]. In the 1H NMR spectrum of SACD, the -CH_2_ proton in the SA group corresponds to the proton peak at 2.70~2.54 ppm, indicating that SA has successfully grafted onto the β-CD molecule. It can be seen from SACD and EO/SACD that the peaks of H-2, H-3, H-4, H-5, and H-6 have shifted and broadened [37]. Similarly, Hartell et al. also observed broadening of the peaks after the inclusion of β-cyclodextrin with artesunate [38]. It can be explained that inclusion has occurred.

The DS was calculated from the ^1^H NMR spectrum by comparing the integrated signal area of the methylene protons of the succinyl group (2.5–2.6 ppm) to that of the anomeric protons (H-1) of the β-CD glucopyranose units (4.8–5.0 ppm). The resulting DS value was 2.31 ± 0.07 [39]. This value indicates that, on average, four succinic acid molecules were grafted onto each cyclodextrin molecule. This result is consistent with previous studies which confirm that succinic acid undergoes monoesterification rather than cross-linking with cyclodextrin [40]. Water solubility of β-CD is enhanced by the substitution of its hydroxyl groups, which disrupts the intramolecular hydrogen bonds between C-2 and C-3 positions [41].

#### 3.1.4. X-Ray Diffraction Testing

**Figure 3:**
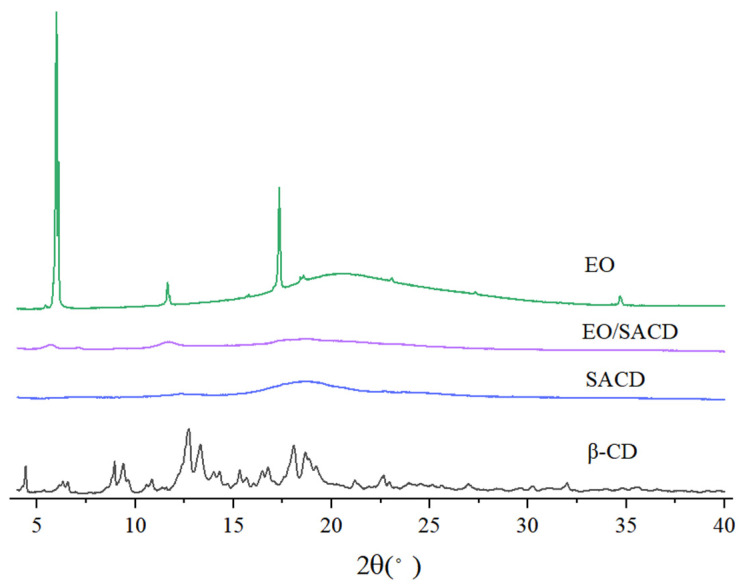
The XRD spectra of β-CD, SACD, EO and EO/SACD.

As shown in Figure 3, native β-CD exhibits a highly dense crystalline structure. In contrast, SACD displays significantly fewer diffraction peaks, indicating a reduction in crystallinity due to the reaction with SA [42]. Notably, the crystalline structure of SACD is completely absent after esterification, suggesting a transition to an amorphous phase. The introduction of SA moieties in place of hydroxyl groups on β-CD disrupts its crystalline structure, primarily by preventing the establishment of hydrogen bonds within and between molecules [43].

The X-ray diffractogram of EO reveals distinct peaks at 5.99°, 11.66°, and 17.33°, characteristic of its crystalline nature. However, the EO/SACD inclusion complex exhibits an XRD pattern nearly identical to that of amorphous SACD, with no detectable EO-derived peaks. This confirms that the encapsulated EO exists in an amorphous state within the SACD matrix. For biologically active molecules, the amorphous state typically requires less energy to disrupt intermolecular interactions, which frequently results in enhanced solubility [44]. The development of EO/SACD complexes enables EOs to remain in an amorphous state, thereby significantly improving their solubility in aqueous systems, a key factor in enhancing the bioavailability of EOs.

#### 3.1.5. Thermogravimetric Analysis

**Figure 4:**
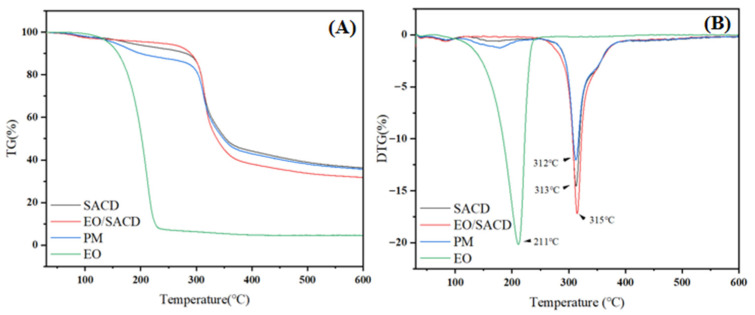
The TG (**A**) and DTG (**B**) curves of SACD, EO, EO/SACD and physical mixture of EOs (PM) and SACD.

As shown in Figure 4A, all samples experienced relatively slow mass loss during the initial stage of heating, while mass loss accelerated rapidly when the temperature reached higher levels. The slow mass loss during the initial stage was primarily due to the elimination of moisture from the specimens. The subsequent rapid mass loss observed at elevated temperatures resulted from the thermal decomposition of the molecular framework [45]. As shown in Figure 4A, the free cinnamon EO experienced a sharp weight loss (95.41%) between 40 °C and 230 °C, indicative of its inherent volatility and low thermal stability [46]. In stark contrast, the EO/SACD inclusion complex exhibited markedly improved stability, with only 3.94% weight loss in the corresponding temperature range (40–245 °C). This dramatic enhancement can be attributed to the molecular encapsulation of EO constituents within the SACD cavity. It physically shields the volatile guest molecules, thereby suppressing their evaporation and thermal degradation at lower temperatures.

Furthermore, the stabilization mechanism is corroborated by the DTG analysis (Figure 4B). The maximum decomposition temperature of the EO/SACD complex (315 °C) was higher than that of the EO (211 °C) and the PM (312 °C). This positive shift in temperature for the inclusion complex suggests that the guest–host interactions between EO and SACD—potentially through hydrogen bonding or van der Waals forces—impart additional stability to the molecular framework, requiring higher energy input for decomposition. Therefore, the superior thermal stability of EO/SACD is a direct consequence of successful cavity inclusion, which provides a protective barrier and stabilizing molecular interactions.

#### 3.1.6. Electron Microscope

**Figure 5:**
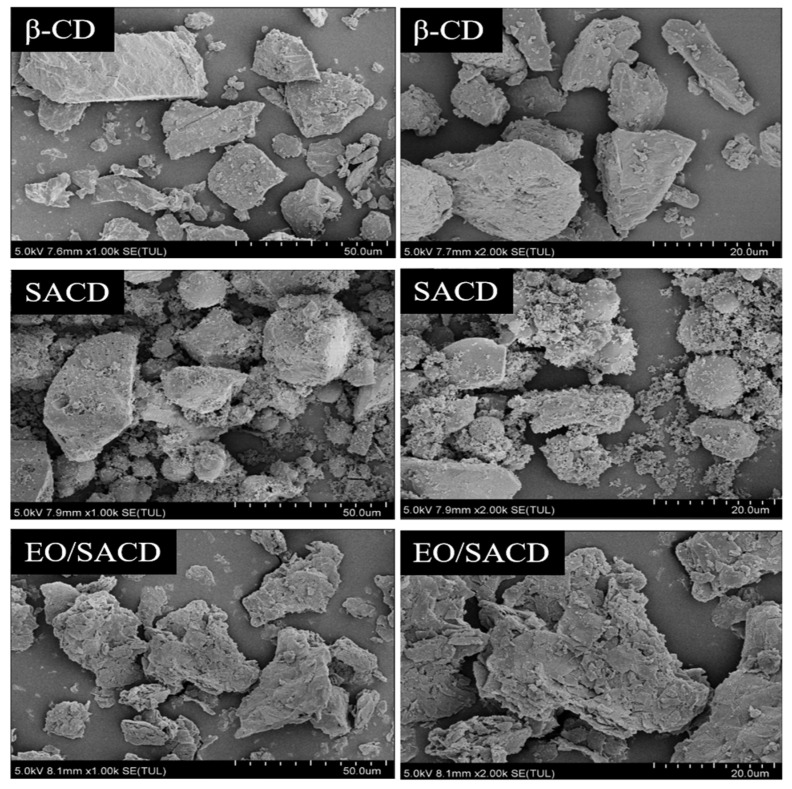
The SEM images of β-CD, SACD and EO/SACD.

As shown in Figure 5, unreacted β-CD exhibits an irregular block-like morphology with an uneven surface, wrinkles, and sharp edges [47]. After esterification, the cyclodextrin SACD undergoes significant changes, with uneven particle sizes and a tendency for particles to aggregate into clusters [48]. The crystalline structure partially disappears, giving rise to spherical structures, and SACD adopts an amorphous structure. The SEM image of EO/SACD exhibits a structure resembling broken tiles, showing a marked difference from the SEM image of SACD [49]. Aggregation phenomena have disappeared, and the structure is sharper and more compact. The microstructure of EO/SACD has undergone significant changes, indicating that EO has been encapsulated.

#### 3.1.7. Molecular Docking

**Figure 6:**
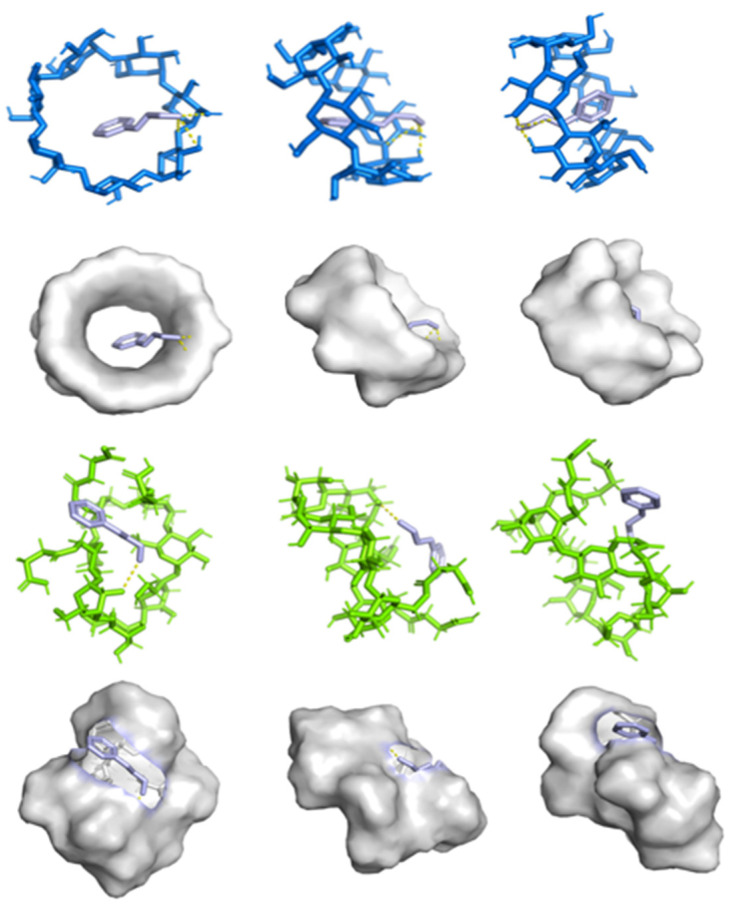
Docking conformations of β-CD (blue) and SACD (green) with cinnamaldehyde (purple).

Molecular docking enables the prediction of binding affinity between hydrophobic guest molecules and cyclodextrins, allowing identification of the most stable configuration for the host–guest complex. As shown in Figure 6, it can be observed that cinnamaldehyde molecules enter the hydrophobic cavities of β-CD and SACD through their broad ends via hydrogen bonding interactions, with minimum binding energies of 3.6 kcal/mol and 3.4 kcal/mol, respectively. This indicates the formation of a stable inclusion complex, whose stability arises primarily from the formation of hydrogen bonds. Notably, our molecular simulation results are consistent with the experimental data obtained earlier. The binding energy of SACD after embedding essential oil is negative, indicating that the binding is stable [48].

### 3.2. Antioxidant Properties and In Vitro Sustained Release

#### 3.2.1. Antioxidant Activity

**Figure 7:**
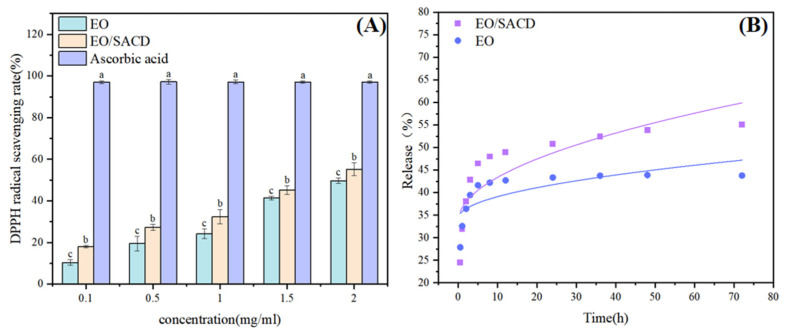
DPPH radical scavenging rate (**A**), in vitro sustained release of EO/SACD (**B**). Note: all above data were mean ± standard error. a–c the different letters mean the significant difference (*p* < 0.05).

Figure 7A presents the antioxidant activity of EO and the EO/SACD against DPPH radicals, evaluated using ascorbic acid as a control across a concentration range of 0.10–2 mg/mL Both samples demonstrated concentration-dependent DPPH radical scavenging effects. The EO/SACD complex demonstrated significantly stronger antioxidant activity than free EO, as evidenced by its significantly lower IC_50_ value (2.052 mg/mL vs. 2.297 mg/mL; *p* < 0.05). Overall, the inclusion complex showed stronger antioxidant activity than pure EO (*p* < 0.05). The observed enhancement is likely attributable to the enhanced stability and solubility of EO within the inclusion complex, which may facilitate greater radical scavenging capacity [23]. Thus, the supramolecular inclusion strategy not only improves EO’s stability and water solubility but also enhances its antioxidant performance.

#### 3.2.2. Analysis of Release Rate

Figure 7B illustrates the in vitro release profile of cinnamon essential oil from the EO/SACD inclusion system over time. It is evident that the release rate of EO/SACD is higher than that of EO, which can be attributed to improved solubility [50]. Relevant studies indicate that the in vitro sustained-release trend of lipophilic molecules encapsulated in cyclodextrin is consistent with the findings of this experimental study. The rapid release is attributed to the desorption of free essential oil adsorbed on the particle surface. Subsequently, the encapsulated oil is released primarily through diffusion from the cyclodextrin cavities and surface. According to the Higuchi fitting model, the inclusion compound follows the equation y = 3.09 x^1/2^ + 33.614 (R^2^ = 0.665); and for essential oil, y = 1.52 x^1/2^ + 34.314 (R^2^ = 0.505), indicating that inclusion compounds follow a diffusion-controlled mechanism more closely than free essential oil. Temperature affects molecular kinetic energy; high temperatures typically accelerate the dissociation of host-guest complexes and the diffusion rate of released essential oils. Diffusion occurs more effectively under neutral pH conditions [51]. This demonstrates that the SACD carrier plays a crucial role in regulating and sustaining essential oil release. Relevant studies indicate that the in vitro sustained-release trend of lipophilic molecules encapsulated in cyclodextrin is consistent with the findings of this experimental study [52].

### 3.3. Applications in Fish Meat

#### 3.3.1. Drip Loss Rate Measurement

**Figure 8:**
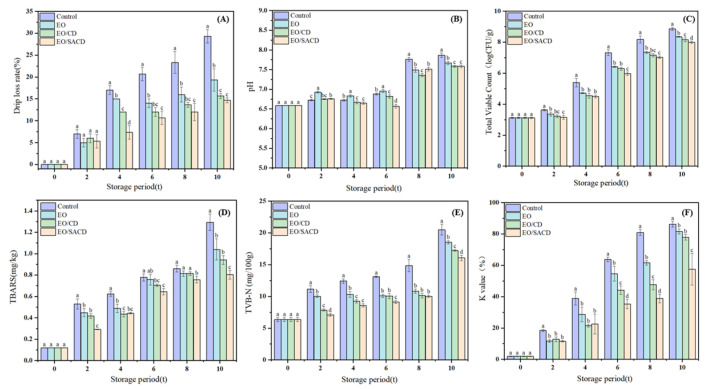
Changes in quality indicators of grass carp during storage at 4 °C for 0–10 days. (**A**) Drip rate, (**B**) pH, (**C**) Total volatile hydrocarbons, (**D**) Peroxide value, (**E**) Total volatile basic nitrogen, (**F**) K value. Note: all above data were mean ± standard error. a–d the different letters mean the significant difference (*p* < 0.05).

Figure 8A illustrates the effects of different treatment methods on the drip loss rate of grass carp during cold storage. All experimental groups exhibited a gradual increase in drip loss rate over time, with the control group consistently demonstrating higher values than the treated groups. Similar results were found in other research [53]. As storage duration extended, microbial spoilage activity intensified in the later stages, resulting in the formation of yellow surface mucus and a concomitant sharp increase in drip loss. Notably, the drip loss rate was markedly decreased in the encapsulation treatment group relative to the EO group. Furthermore, SACD more effectively reduced drip loss than unmodified β-CD (*p* < 0.05, η^2^ = 0.96). This difference may be attributed to the superior antibacterial activity of SACD, which exhibits higher solubility than β-CD, thereby more efficiently inhibiting microbial proliferation on the fish surface.

#### 3.3.2. Determination of pH

Figure 8B demonstrates that the pH values on the 4th day exhibit a downward trend. This initial decline can be attributed to the formation of organic acids, including lactic acid, generated via glycolytic processes in fish muscle [31]. The magnitude of this decrease is influenced by the glycogen content in the muscle tissue. However, as storage progresses, microbial growth leads to protein degradation and the production of alkaline substances—including ammonia and trimethylamine that raise the pH over time [54]. It should be noted that pH is affected by multiple factors, including packaging method, fish species, and growth environment. Therefore, it cannot serve as a precise indicator of freshness but rather as an auxiliary parameter. In the final stage of storage, the pH of the control group exhibited a markedly higher value than that of the treated group (*p* < 0.05, η^2^ =0.92). This difference suggests that both free essential oils and their inclusion complexes suppressed microbial growth, leading to fewer alkaline breakdown products.

#### 3.3.3. Total Viable Count (TVC)

As shown in Figure 8C, during storage, the total number of colonies showed a gradual increase [55]. During the same storage period, the total colony count in the control group demonstrated a statistically significant increase compared to the remaining treatment groups (*p* < 0.05, η^2^ = 0.98). This difference can be attributed to the antibacterial properties of EOs, which slow down microbial proliferation in fish fillets. During the later storage period, the EO/CD group exhibited higher total colony counts compared to the EO/SACD group. Moreover, on Day 6, the total colony count of EO/SACD was significantly lower than that of other groups (*p* < 0.05, η^2^ = 0.97), indicating that the modified encapsulation exhibits superior antibacterial properties. This observation suggests that encapsulation with cyclodextrin not only reduces the evaporation and oxidation of EOs but also enables a sustained release effect, thereby more effectively inhibiting microbial growth in fish.

#### 3.3.4. Determination of TBARS

Grass carp contains a high proportion of unsaturated fatty acids, which are highly susceptible to oxidation and hydrolysis due to exposure to microorganisms and air [56]. Figure 8D demonstrates that the TBARS levels exhibited a general upward trend for the duration of storage. Both the EO and the inclusion complex groups showed lower values compared to the control, indicating that both the EO and its complexes can suppress TBARS values in fish meat and thus delay lipid oxidation. Additionally, the antioxidant efficacy of the complex was superior to that of the EO alone. Specifically, the EO/SACD group exhibited higher antioxidant activity than the EO/CD group.

#### 3.3.5. Determination of TVB-N

As shown in Figure 8E, the TVB-N values of fish fillets increased significantly with prolonged storage time, reflecting progressive spoilage. According to regulatory standards, the secondary freshness threshold for fish is TVB-N ≤ 20 mg N/100 g. The results indicate a statistically significant reduction in the measured values for the treatment group relative to the control (*p* < 0.05, η^2^ = 0.93). All other treatment groups remained below the limit, demonstrating that EO can inhibit the production of volatile nitrogenous compounds. Notably, fish treated with cyclodextrin inclusion complexes exhibited lower TVB-N values than those treated with EO alone. The stability and controlled release of active compounds in cinnamon essential oil are significantly enhanced through cyclodextrin encapsulation, thereby improving its preservative effect [57]. At 0–6 days and on day 10, TVB-N levels in the EO/SACD group were even lower than those in the EO/CD and EO groups (*p* < 0.05, η^2^ = 0.94), indicating that structural modification of cyclodextrin further enhanced its ability to inhibit putrefactive nitrogen compounds.

#### 3.3.6. Determination of K Value

With prolonged storage, the K-values of all fish samples demonstrated a consistent upward trend. K-values between 20% and 50% indicate secondary freshness, suitable for processing raw materials; values between 60% and 80% indicate initial spoilage; values above 80% indicate spoiled fish meat [58]. As shown in Figure 8F, on day 4, all treatment groups exhibited markedly reduced K-values compared to the control (*p* < 0.05, η^2^ = 0.78), remaining within the range appropriate for raw material processing. By day 6, the control group had reached the spoilage stage, whereas the treated groups had not yet entered this phase (*p* < 0.05, η^2^ = 0.95). Based on the experimental results, the EO/SACD group exhibited the most pronounced efficacy in extending the shelf life of grass carp, followed by the EO/CD group, and finally the EO group. The modified cyclodextrin inclusion complex demonstrates a significant efficacy in enhancing the freshness preservation of fish meat.

#### 3.3.7. Texture Analysis

**Figure 9:**
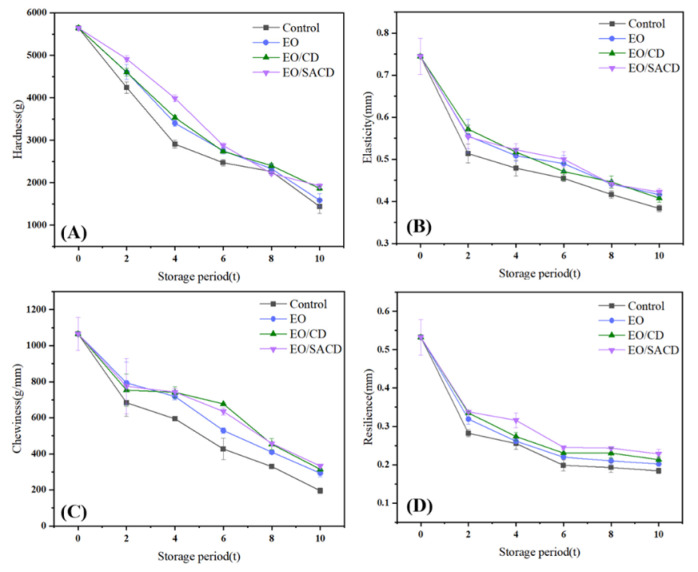
Changes in Textural Properties of Grass Carp During Storage: Hardness (**A**); Elasticity (**B**); Chewiness (**C**); Resilience (**D**).

The textural deterioration of fish meat during storage, characterized by a loss of hardness, springiness, chewiness, and resilience, is primarily attributed to the synergistic action of microbial proliferation and endogenous enzymatic activity. These biological processes progressively degrade the structural proteins of the muscle tissue, leading to a breakdown of its structural integrity [59]. As shown in Figure 9, a general reduction in the hardness, springiness, chewiness, and toughness of fish meat was observed across all treated groups relative to the control during the storage period. From days 0 to 4, the EO/SACD group showed significantly higher hardness than the other groups (*p* < 0.05); however, its hardness decreased below that of the other groups by day 8. On day 10, the EO/SACD and EO/CD groups demonstrated significantly greater hardness compared to the remaining groups (*p* < 0.05, η^2^ = 0.85). In terms of chewiness, the EO/CD and EO/SACD groups demonstrated a statistically significant increase over the other treatments at day 6 (*p* < 0.05, η^2^ = 0.93). On day 4, fillets treated with EO/SACD exhibited markedly greater toughness compared to all other groups (*p* < 0.05, η^2^ = 0.82). Overall, the toughness decreased in the following order: EO/SACD > EO/CD > EO > control. These results indicate that the encapsulation approach effectively mitigates microbial degradation of tissue structures, thereby preserving the textural quality of fish fillets.

## 4. Conclusions

This study successfully synthesized SACD to encapsulate EO, mitigating its limitations of high volatility, poor water solubility, and strong odor. During refrigerated storage at 4 °C of grass carp meat, the EO/SACD composite exhibited superior preservation effects over free EO and the control, as evidenced by significantly lower TVB-N, TBARS, and total viable count (*p* < 0.05), together with improved texture parameters. It also demonstrated stronger in vitro antioxidant capacity with a lower IC_50_ value compared to free EO. The EO/SACD complex effectively enhanced grass carp preservation by inhibiting microbial proliferation, suppressing lipid oxidation, and delaying protein degradation. A limitation of this study is the focus on a single fish species and the lack of characterization of the physical mixture control. Future work should explore applications in other aquatic products, investigate the in vivo safety of SACD, and include an economic analysis for scale-up. This study confirms that cyclodextrin encapsulation mitigates key drawbacks of free EOs, offering a promising and effective strategy for extending grass carp shelf life.

## Figures and Tables

**Table 1:** MIC Values of Different Essential Oils Against Various Bacteria.

Microbial Strain	Cinnamon EO	Oregano EO	Lemongrass EO	Thyme EO
*Escherichia coli*	1.25 mg/mL	>2.5 mg/mL	>2.5 mg/mL	>2.5 mg/mL
*Staphylococcus aureus*	1.25 mg/mL	>2.5 mg/mL	>2.5 mg/mL	>2.5 mg/mL
*Pseudomonas fluorescens*	2.5 mg/mL	>2.5 mg/mL	>2.5 mg/mL	>2.5 mg/mL
*Listeria monocytogenes*	2.5 mg/mL	>2.5 mg/mL	>2.5 mg/mL	>2.5 mg/mL

## Data Availability

The original contributions presented in the study are included in the article; further inquiries can be directed to the corresponding author.
